# Malignant Spinal Cord Compression Syndrome as an Initial Presentation of Testicular Cancer

**DOI:** 10.1155/2018/5757434

**Published:** 2018-10-08

**Authors:** Carlos Eduardo Salazar-Mejía, Edio Llerena-Hernández, David Hernández-Barajas, Oscar Vidal-Gutiérrez, Adriana González-Gutiérrez, Rolando Jacob Martínez-Granados, Blanca Otilia Wimer-Castillo

**Affiliations:** Centro Universitario Contra el Cáncer, University Hospital “Dr. José Eleuterio González” and Faculty of Medicine, Universidad Autónoma de Nuevo León, Monterrey, Nuevo León, Mexico

## Abstract

Malignant spinal cord compression syndrome (MSCCS) occurs in 2.5 to 5% of all oncological patients. In 20% of the cases, it is the initial manifestation. This syndrome is a rare event among germ cell tumors (GCT), occurring in only 1.7% of the patients. We present the case of a 24-year-old man who arrived at the emergency department with dysesthesia and paraparesis as well as urinary incontinence. Imaging studies showed an infiltrative lesion in the left testicle, pulmonary and hepatic metastatic disease, and a large retroperitoneal ganglionar conglomerate that infiltrated the spinal cord through the intervertebral foramina of the vertebra level T11 with displacement of the L1 vertebral body. A postoperative biopsy showed a pure embryonal carcinoma. In the initial approach of a young man who presents spinal cord compression, the presence of MSCCS associated with GCT should be considered as a possible cause. A high level of suspicion is required to achieve a timely diagnosis, to grant the patient the best possible outcome.

## 1. Introduction

Malignant spinal cord compression syndrome (MSCCS) occurs in 2.5 to 5% of all oncological patients. In 20% of the cases, it is the initial manifestation [1]. Detection and intervention within the first 24 hours are essential for therapeutic success [2]. Although several case reports have documented the presence of MSCCS in patients with germ cell tumors (GCT) as an initial presentation or as part of their evolution after primary therapy, there is little evidence on the best therapeutic option in this context. We present a rare case of MSCCS as the initial presentation of a pure testicular embryonal carcinoma.

## 2. Case Presentation

A 24-year-old man arrived at the emergency department with a four-month history of pain and swelling of the left testicle. A week ago, he developed progressive edema in the lower limbs, which was followed 48 hours before admission by dysesthesia and paraparesis as well as urinary incontinence. He had no previous medical history and did not consume alcohol or use any illicit drug or medication.

Physical examination on admission showed a blood pressure of 100/60 mmHg, a temperature of 36°C, a pulse rate of 90/min, and a respiratory rate of 22/min; his height was 1.75 m, weight 98 kg, and BMI 32 kg/m^2^. He referred bilateral paresthesia of the lower limbs and pain on mobilization of the lumbar spine. The left scrotal sac was enlarged and indurated, and there was a mass in the left scrotum that was indistinguishable from the right testis and provoked displacement of the penis and right testis. No inguinal lymphadenopathy was identified. When examining both lower extremities, we found edema without fovea. Neurological examination revealed hypoesthesia, areflexia, and decreased muscle strength.

Laboratory tests revealed normal liver function. Hemoglobin was 9.13 g/dL, MCV was 87.9 fL, WBC was 11.9 K/*μ*L, neutrophil count was 9.81 K/*μ*L, lymphocyte count was 1.59 K/*μ*L, and the platelet level was 252 K/*μ*L. Serum glucose was 148 mg/dL, BUN was 38 mg/dL, creatinine was 1.3 mg/dL, and calcium was 8.9 mg/dL. Serum alpha-fetoprotein (AFP) was 11.28 ng/mL, lactate dehydrogenase was 1687 U/L (normal range 91–180 IU/L), and human chorionic gonadotropin level was 10.05 IU/mL.

A scrotal ultrasound showed a hypoechoic oval-shaped infiltrative lesion in the left testicle of 6.1 × 3.2 × 2.7 cm, associated with a large left hydrocele, with an estimated volume of 450 mL. Microcalcifications were seen in both testis.

A contrasted CT scan of the thorax, abdomen, and pelvis showed bilateral supraclavicular adenopathies and multiple round pulmonary nodules with well-defined edges of bilateral and diffuse distribution. In the liver, there were two hyperdense lesions: one larger lesion of 4.8 × 2 cm located in hepatic segment IV and a second lesion of 1.9 cm in segment VIII, with partially defined borders and enhancement to contrast administration. There was also a large retroperitoneal ganglionar conglomerate of 10 × 6 cm, which infiltrated the spinal cord.

**Figure 1:**
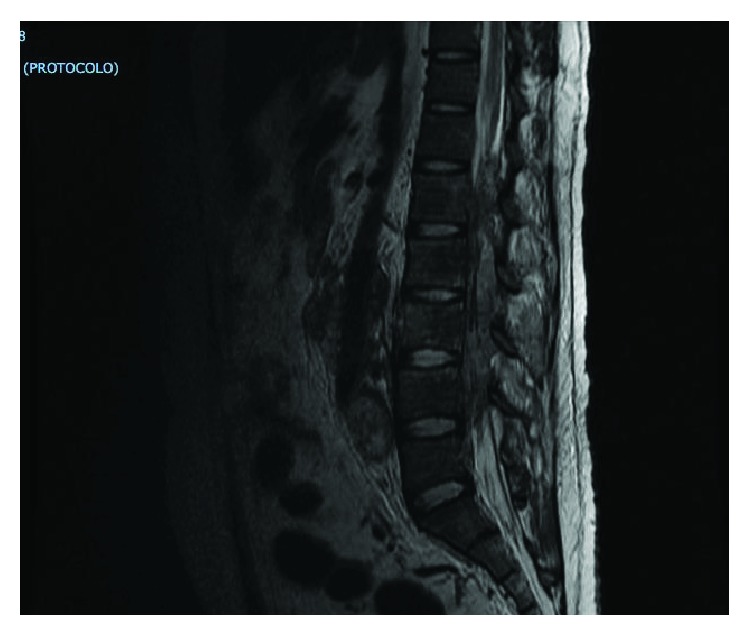


**Figure 2:**
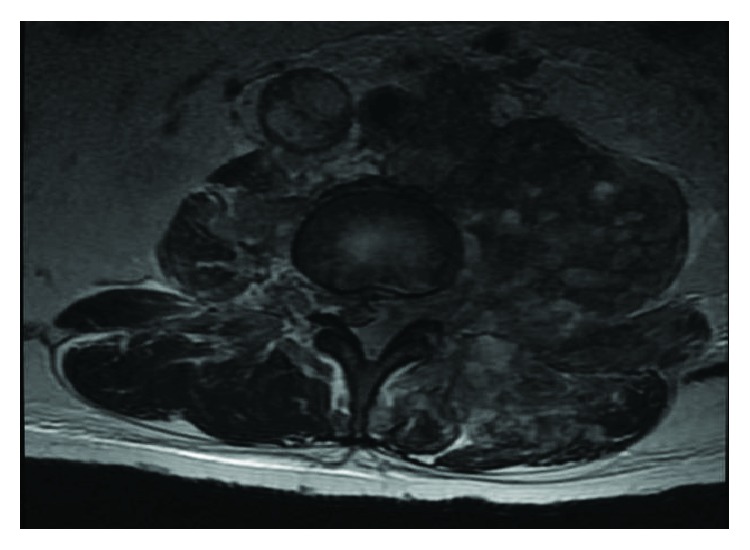


An MRI of the dorsal and lumbar spine showed a large and heterogeneous retroperitoneal conglomerate which invaded the left psoas muscle and infiltrated the spinal cord through the intervertebral foramina of T11 with displacement of the L1 vertebral body (Figure 1). Metastatic infiltration of the vertebral bodies L3–L5 was also seen (Figure 2).

A diagnosis of clinical stage IIIC testicular cancer was established with a poor prognosis due to nonpulmonary visceral metastases. After initial treatment with high-dose intravenous corticosteroids, the case was evaluated by a multidisciplinary oncological team. Three fractions of external beam radiotherapy were given. He then underwent a left radical orchiectomy by an inguinal approach with a left hemiscrotectomy, without complications. A postoperative biopsy showed a pure embryonal carcinoma with invasion of the spermatic cord and necrosis of more than 50% of its surface. After finishing 10 fractions of radiotherapy for a total of 30 Gy, and improving his renal function with intravenous crystalloids, the patient received his first cycle of chemotherapy (CT). We planned a CT regimen based on bleomycin, etoposide, and cisplatin (BEP), repeated every 21 days for a total of four cycles.

After the eighth day of CT, the patient presented sudden dyspnea at rest that progressed to hypoxemic respiratory failure. Due to this condition, we decided to perform orotracheal intubation and the patient was then transferred to the intensive care unit. An angio-CT revealed the presence of bilateral pulmonary thromboembolism. Despite the treatment received, the patient died three days later.

## 3. Discussion

MSCCS is a devastating manifestation of metastatic cancer that results from compression of the dural sac or its contents by an extradural tumoral mass that manifests clinically by local or radicular pain in 83% to 95% of the cases; this is usually the initial symptom [1]. The presence of motor deficit that incapacitates deambulation is manifested in 50 to 68% of the cases, while sensory or autonomic deficit can be observed in about 60% of these patients [3]. The main oncological pathologies associated with MSCCS are lung (22.9%), breast (19.9%), and prostate cancer (18.4%) [4]. MSCCS is a rare event among GCT, occurring in only 1.7% of the patients [5].  Table 1 summarizes the reported cases of MSCCS as an initial presentation of GCT in the English literature.

GCT are the most common malignancies in men aged 15 to 39 years. Their main origin is the gonads; extragonadal GCT comprise only 5 to 10% of the cases [6]. Within the histological classification of GCT, pure embryonal carcinoma comprises approximately 2% of the cases [7]. Characteristically, these tumors generally predispose only to modest elevation of the AFP [8].

Independent adverse factors in GCT include the degree of elevation of tumor markers, mediastinal primary site, and the presence of nonpulmonary visceral metastases. Regardless of the initial clinical stage and risk stratification, the therapeutic goal is to achieve cure of the disease, since patients with advanced GCT who survive and remain disease-free for more than 2 years after their diagnosis have a great chance of surviving in subsequent years [9]. The standard of treatment for patients with advanced disease and intermediate to high risk, is a CT regimen based on BEP every 21 days for 4 cycles [6, 10].

The management of a patient with MSCCS must be multidisciplinary and integral, taking into account symptomatic control and the primary tumor, as well as possible associated comorbidities. MRI is the imaging study of choice to better characterize the degree of involvement of the spinal cord canal. Initial treatment includes the use of high doses of intravenous corticosteroids and decompression by surgery and/or radiotherapy (RT) [11, 12]. The ASTRO guidelines for palliative RT for bone metastases, in its update of 2017, recommend doses of 8 Gy in a single fraction (Fx), 20 Gy/5 Fx, 24 Gy/6 Fx, or 30 Gy/10 Fx for patients with bone disease not previously treated [13]. CT is the main therapeutic option in GCT, due to the high chemosensitivity exhibited by these tumors [14]. The key prognostic factor for optimal functional outcome is the degree of functional capacity at the time of treatment initiation [3].

The benefit of adding radiotherapy prior to chemotherapy (RT/CT) in this group of patients has been debated for decades. The largest series in this regard is a retrospective analysis of the database of patients with GCT of the Memorial Sloan-Kettering Cancer Center from 1984 to 2009 [5]. Of 1734 patients included, 29 were found to have MSCCS (10 patients with seminoma, 19 nonseminoma). The main spinal cord compression sites were located in the lumbar and thoracic spine and 20% showed involvement in multiple sites. The most used CT regimen was BEP in both groups, and 62% of the patients had not previously received CT. This study showed no difference in symptomatic response between both treatment arms. A significant difference was found in overall survival after diagnosis and first-line treatment of MSCCS between the group treated with RT/CT (*n* = 4) and the group with CT alone (*n* = 11), with a mean overall survival not reached in the CT group versus 22 months in the RT/CT group (*p* = 0.04). An analysis of patients who received 2 or less lines of treatment showed a mean survival of 138 months for the CT group alone compared to 38 months in the RT/CT group (*p* = 0.23).

Based on the results of this analysis, CT could be considered as an appropriate treatment option in patients with newly diagnosed GCT who present MSCCS, as well as those with no more than two previous treatment regimens if the disease is chemosensitive. In patients who have received 2 or more cycles of CT, RT/CT may be an option [5]. Despite the limitations of this study (retrospective design and number of patients included), it is unlikely that a prospective protocol will be carried out in this regard, given the rarity of the presentation.

The event that predisposed to the clinical deterioration and subsequent death of our patient was the development of pulmonary thromboembolism. Cancer patients on active therapy have a higher risk for venous thromboembolism (VTE) and those receiving CT account for as much as 13% of the total burden of VTE. The presence of this complication is strongly associated with an increased early all-cause mortality during the course of CT and reduced long-term survival [15].

Within the initial approach of a young man who presents with spinal cord compression syndrome, the presence of MSCCS associated with GCT should be considered as a possible cause. A high level of suspicion and a complete physical examination, including a genital exam, is required to achieve a timely diagnosis, in order to grant the patient the best possible outcome.

## Figures and Tables

**Table 1:** Summary of the reported cases of MSCCS as an initial presentation of GCT in the English literature.

Author/year	Country	Age of presentation	Mechanism of spinal cord injury	Spinal level of metastasis causing cord compression	Histology	Treatment of MSCCS, in chronological order	Outcome, follow-up
Nelson and Ruffolo 1966 [16]	USA	63 years	Extradural compression	T9	Classic seminoma	Laminectomy	Death, 12 days
Arnold et al. 2000 [17]	USA	41 years	Extradural compression	T7	Mixed GCT	Laminectomy, unspecified CT	Good, 1 year
Yee et al. 2007 [18]	Canada	44 years	Extradural compression	T4	Classic seminoma	Steroids, EBRT 20 Gy/5 Fx, BEP	Good, 1 year
Salazar-Mejía et al. 2018	Mexico	24 years	Extradural compression	T11	Pure embryonal carcinoma	Steroids, EBRT 30 Gy/10 Fx, BEP	Death, 3 weeks

CT, chemotherapy; BEP, bleomycin/etoposide/cisplatin; EBRT, external beam radiotherapy.
